# Downregulation of monocyte miRNAs: implications for immune dysfunction and disease severity in drug-resistant tuberculosis

**DOI:** 10.3389/fimmu.2023.1197805

**Published:** 2023-06-29

**Authors:** Pavithra Sampath, Manju Moorthy, Athul Menon, Lekshmi Madhav, Aishwarya Janaki, Madhavan Dhanapal, Alangudi Palaniappan Natarajan, Syed Hissar, Uma Devi Ranganathan, Gopalakrishna Ramaswamy, Ramalingam Bethunaickan

**Affiliations:** ^1^ Department of Immunology, Indian Council of Medical Research (ICMR)-National Institute for Research in Tuberculosis (NIRT), Chennai, India; ^2^ TheraCUES Innovations Pvt. Ltd, Bangalore, India; ^3^ Department of Clinical Research, ICMR-National Institute of Research in Tuberculosis (NIRT), Chennai, India

**Keywords:** monocyte miRNA, drug-resistant -TB, immune dysfunction, *Mycobacterium tuberculosis*, NanoString, monocyte sorting

## Abstract

**Background:**

Monocyte miRNAs govern both protective and pathological responses during tuberculosis (TB) through their differential expression and emerged as potent targets for biomarker discovery and host-directed therapeutics. Thus, this study examined the miRNA profile of sorted monocytes across the TB disease spectrum [drug-resistant TB (DR-TB), drug-sensitive TB (DS-TB), and latent TB] and in healthy individuals (HC) to understand the underlying pathophysiology and their regulatory mechanism.

**Methods:**

We sorted total monocytes including three subsets (HLA-DR^+^CD14^+^, HLA-DR^+^CD14^+^CD16^+^, and HLA-DR^+^CD16^+^cells) from peripheral blood mononuclear cells (PBMCs) of healthy and TB-infected individuals through flow cytometry and subjected them to NanoString-based miRNA profiling.

**Results:**

The outcome was the differential expression of 107 miRNAs particularly the downregulation of miRNAs in the active TB groups (both drug-resistant and drug-sensitive). The miRNA profile revealed differential expression signatures: i) decline of miR-548m in DR-TB alone, ii) decline of miR-486-3p in active TB but significant elevation only in LTB iii) elevation of miR-132-3p only in active TB (DR-TB and DS-TB) and iv) elevation of miR-150-5p in DR-TB alone. The directionality of functions mediated by monocyte miRNAs from Gene Set Enrichment Analysis (GSEA) facilitated two phenomenal findings: i) a bidirectional response between active disease (activation profile in DR-TB and DS-TB compared to LTB and HC) and latent infection (suppression profile in LTB vs HC) and ii) hyper immune activation in the DR-TB group compared to DS-TB.

**Conclusion:**

Thus, monocyte miRNA signatures provide pathological clues for altered monocyte function, drug resistance, and disease severity. Further studies on monocyte miRNAs may shed light on the immune regulatory mechanism for tuberculosis.

## Introduction

Tuberculosis (TB) is caused due to the historical agent *Mycobacterium tuberculosis* (MTB) (1) which possesses a never-ending infection trend and poses a continuous threat to humans. TB management is still uncertain even in this era of advanced medicine. Improved understanding of the disease behavior and identification of stage-specific biomarkers are the pivotal goals of TB research to move forward toward global elimination. The co-evolution of MTB towards compatible human macrophage habitats (1) allowed them to launch a wide presentation of clinical infection, including clearance, low-grade TB (2), disease progression (3), and drug resistance. The prolonged latency state within the human host signifies their survival rate and re-infecting ability (4). However, evidence in the literature has put forward the theory that the active disease progression duration from latent infection (LTB) is within the initial two years after exposure (5, 6). Epigenetic drug tolerance is another phenomenon in which MTB can tolerate antibiotics through metabolic shutdown and acquire a non-replicating latent state (7, 8). Among people with latent infections, those with incipient or sub-clinical infections are at higher risk towards progressing to active disease (3, 9, 10). The available tests for LTB identification through immunological response such as the tuberculin skin test (TST) and an interferon-gamma release assay (IGRA) can neither detect the ongoing infection nor predict the risk of disease progression (11). Biomarkers for identifying high-risk groups are essential for considering them as the primary target for TB preventive therapy rather than the whole group of latently infected individuals. This will enable the wise usage of antibiotics and minimalize the emergence of drug resistance in the future. Transcriptomic signatures have gained attention over immunological assays in distinguishing the LTB individuals with viable bacilli from those who have eliminated the pathogen (12, 13).

A deeper understanding of the disease’s behavior and pathogenesis has uncovered more challenges for TB research toward elimination (14). An interesting and shocking observation from TB aerobiology studies was the occurrence of MTB transmission even with tidal breathing (15). This is even more dangerous than the pre-existing facts and can severely undermine the diagnosis of asymptomatic and culture-negative individuals. Another critical challenge for TB control and treatment is the emergence of intrinsic and extrinsic drug resistance in MTB (7). Studies postulate that extrinsic resistance occurs through chromosomal mutations (16), continuous exposure to drugs, modified drug targets (17), and compensatory evolution and epistasis (18–20). Mechanisms such as cell envelope impermeability, drug efflux, drug degradation and modification, and target mimicry contribute to intrinsic resistance in MTB (17, 21). Long non-coding RNAs are one major contributor to the pathological progression of MDR-TB (22). The infection loop and varied spectrum contribute to the complex nature of the disease and ultimately affect the overall treatment quality and impede the diagnosis with limited high-cost assays. 

However, the human immune system responds to MTB in different ways and is self-sufficient to deliver protection against TB. The concepts of trained immunity, autophagy, and epigenetic programming expose the strength of innate cells, particularly macrophages, for early clearance of MTB infection (23). In contrast, innate immune manipulation by MTB molecules leads to failure to control inflammation (24) and accelerates infection chronicity. The paradoxical behavior of host innate cells that permit bacterial growth and immune evasion is still unknown. Transcriptomics has unveiled the dominance of myeloid cell gene signatures and their immune regulation during TB (25). Chiefly, peripheral monocyte abundance and dysfunction are noted to be linked with innate immune activation through the enhanced expression of genes involved in the anti-microbial effect, inflammatory markers, and chemokines that progress alongside TB disease severity (26–28). Beyond RNAs, the regulation of monocyte mechanisms and their heterogenous response to the mycobacterium is credited to short, non-coding microRNAs (miRNAs). The functions of miRNAs are wide-ranging with some being reported to be involved in gene silencing, immune modulation, and the pathogenesis of infectious diseases, particularly TB (29, 30). The multifaceted effect of monocyte miRNAs in host killing machinery, signaling pathways, cytokine production, and their biomarker potency through differential expression are reviewed in our previous article (31). This review process gave more insight into macrophage miRNAs with limited information about their precursor monocytes. Furthermore, few studies have addressed monocyte miRNA profiling among TB-affected individuals and thus we wanted to know how monocyte-specific miRNAs are expressed across the TB spectrum. We intended to study and understand the monocyte miRNAs from various TB infection states (drug-resistant, drug-sensitive, and latent). We sorted monocytes from peripheral blood mononuclear cells (PBMC) through flow cytometry and subjected them to NanoString-based miRNA profiling. We observed severe downregulation of monocyte miRNAs and their interacting pathways in active TB thus providing a pathological clue for drug-resistant TB.

## Methods

### Study samples

Blood samples from four groups, 1) healthy controls (HC) (n=6), 2) latent TB (LTB) (n=6), 3) drug-sensitive pulmonary tuberculosis (DS-TB) (n=6), and 4) drug-resistant pulmonary tuberculosis (DR-TB) (n=6) were collected with approval from the Institutional Ethics Committee of ICMR-National Institute for Research in Tuberculosis, Chennai (NIRT, IEC 2015022) and from the Greater Chennai Corporation, Tamil Nadu. Adult individuals aged 18-55 years, without other co-morbidities, and who consented were recruited. Healthy volunteers who tested negative for IGRA were regarded as HC (Group 1) and those who tested positive for IGRA were grouped as LTB (Group 2). Those with active TB (DS-TB and DR-TB) were enrolled from primary health care centers of Chennai through regular TB diagnosis. Participants with TB symptoms, confirmed through any one of the TB diagnosis methods (Chest X-ray/Smear/Culture/Gene-Xpert/Clinical presentation) with confirmed sensitivity for first line drugs by sensitivity assays (Drug Sensitivity Test (DST)/Line Probe Assay (LPA)/Gene-Xpert) were categorized as DS-TB (Group 3) and those with confirmed resistance against any of the first line drugs were characterized as DR-TB (Group 4). Baseline samples before the initiation of anti-TB treatment were collected at one time point collection for DS-TB, and DR-TB. For DR-TB, either rifampicin resistance or multi-drug resistance (resistance to both rifampicin and isoniazid) were considered for collection.

### Peripheral blood mononuclear cell isolation

Whole blood (8-12 ml) was collected in heparin vacutainers (BD India Pvt. Ltd., Cat#367874) from study participants and PBMCs were isolated by adopting the protocol from Fuss IJ et al., 2009 (32). Briefly, whole blood was centrifuged at 2600 rpm for 10 minutes to collect the plasma portion. The packed cell volume was diluted with 1X phosphate buffered saline (PBS) (Lonza India Pvt. Ltd. Cat# 17-515F) as per the volume of separated plasma and the initial volume of blood. The PBS-blood mixture was layered on top of the Lymphoprep (STEMCELL Technologies Cat# 07851) gradient solution in 1:1 ratio and centrifuged at 2000 rpm for 30 minutes at 18-20°C without a break to separate the components of blood. The upper layer containing 1X PBS and the remaining cell platelet fraction were removed using the sterile pipet. The mononuclear cells presented as white cloudy layer in between the PBS and Lymphoprep solution was then transferred to fresh tube for further washing procedure. The buffy coat was washed twice with 1X PBS at 1500 rpm for 10 minutes. The final PBMC pellet was suspended with PBS buffer containing 1mM ethylenediaminetetraacetic acid (EDTA) (Himedia Cat# GRM3915) and 2% fetal bovine serum (FBS) (Himedia Cat# RM1112) and the viability of the isolated cells were determined by the trypan blue dye exclusion method.

### Monocyte sorting

Total monocytes were sorted through flow cytometry (FACS Aria-III) (BD, USA) from the PBMCs (approximately 10-15 million cells/ml) by positive selection with monocyte specific fluorochrome tagged antibodies (BD Biosciences and BD Pharmingen). The antibodies used include CD2-FITC (clone: RPA-2.10, BD), CD3-FITC (clone: HIT3a, BD), CD19-FITC (clone: HIB19, BD), CD56-FITC (clone: B159, BD), HLA-DR-PE Cy7 (clone: G46-6, BD), CD14-PE (clone: M5E2, BD), and CD16-BUV737 (clone: 3G8, BD). The stained cells were washed and suspended in 1 to 2 ml of PBS containing EDTA and FBS buffer. FACS Diva software (version 7.0) was used to mark the PBMCs on the FSC-A versus SSC-A plot and single cells were discriminated from doublets using the FSC-A and FSC-H plot. A dump gate was created on the FITC channel which has antibodies for CD2 and CD3-T cells, CD19-B cells, and CD56-NK cells. A FITC negative monocyte population was further ensured with HLA-DR and CD16 antibodies. The HLA-DR single positive cells and HLA-DR+ CD16+ cells accounted for the pure monocyte population. The final gate was plotted with CD14 and CD16 antibodies, where CD14+ cells, CD14+ CD16+ cells, and CD16+ cells were marked together for sorting the pure monocytes. The sorted monocytes count and viability were determined using the trypan blue dye exclusion method. The sorted cells were pelleted at 1500 rpm for 10 minutes and suspended in RLT lysis buffer (Qiagen Cat# 79216) containing 1% of 2-mercaptoethanol (Himedia Cat# MB041) and stored at -80°C.

### RNA extraction and NanoString miRNA expression assay

Total RNA including miRNA was isolated from the sorted monocytes (Range: 1 to 2.8 million cells) in RLT buffer using phenol-chloroform and a Qiagen miRNeasy mini kit (Cat#217004). Cells were received in 150µl of RLT buffer and the volume was made up to 200 µl with RLT buffer and vortexed. Samples were extracted with a standard phenol-chloroform mixture. The aqueous phase obtained after centrifugation was mixed with 700 µl of QIAzol Lysis reagent and mixed well. The lysate was incubated at room temperature for 5 minutes and 140 µl of chloroform was added for phase separation. The rest of the protocol was followed as per the manufacturer’s guidelines. The RNA was eluted in 20µl of nuclease-free water (Ambion, Cat # AM9932). Quantitation was performed using a Qubit RNA HS assay (Invitrogen, Cat # Q32855) kit and also qualitatively analyzed on an Agilent 2100 bioanalyzer nano chip (Agilent, Cat # 5067-1511). Furthermore, 100ng of total RNA was used for the miRNA assay.

With NanoString Human v3A miRNA assay kit (CSO-MIR3-12), miRNA was ligated to mir-Tag with ligation buffer and ligase supplied with the kit. The ligated product was diluted with 15 µl of nuclease-free water, denatured at 85 °C for 5 minutes, and 5 µl of this was hybridized overnight at 65°C with reporter and capture probes. Protocol was followed as per the manual (nCounter miRNA Expression Assay User Manual, MAN-C0009-07). Post hybridization, the samples were analyzed on a NanoString nCounter SPRINT machine.

### miRNA expression analysis

Analysis was performed on all the samples using the nCounter Analysis System (NanoString Technologies) and the nCounter Human v3 miRNA panel with 799 unique clinically relevant miRNA barcodes for endogenous miRNA. The housekeeping genes used in the panel include beta-actin (Actb), beta-2-microglobulin (B2m), glyceraldehyde 3-phosphate dehydrogenase (Gapdh), ribosomal protein L19 (Rpl19), and ribosomal protein lateral stalk subunit P0 (Rplp0). The panel also included the SpikeIn miRNAs, Arabidopsis thaliana miR159a (ath-miR159a), Caenorhabditis elegans (cel)-miR-248 and miR254, Oryza sativa (osa)-miR 414, and miR 442 along with the positive and negative controls for assessing overall assay efficiency as well as for monitoring the ligation efficiency.

The miRNA raw data in.RCC (Reporter Code Count) format was further analyzed using nSolver analysis software (NanoString technologies) version 4.0. The QC metrics were performed as per the guidelines, before proceeding with further analysis. Normalization of the raw data was performed using the geometric mean of the positive controls and the top 100 highly expressed miRNAs (CV% < 50) as per the instructions in the manual (nCounter Data Analysis Guidelines for miRNA (LBL-C0046-01) and nSolver™ 4.0 Analysis Software User Manual (MAN-C0019-08)). The differential expression between the various test and control groups (LTB vs HC, DS-TB vs HC, DS-TB vs LTB, DR-TB vs HC, DR-TB vs LTB, and DR-TB vs DS-TB) were calculated using the build ratio [fold change (FC)] utility present within nSolver. The thresholds considered for classifying significantly differentially expressed miRNAs were, FC >= 1.5 (upregulated) or <= -1.5 (downregulated) with a p-value < = 0.05.

The global miRNA expression level at its normalized state was used to visualize the sample distribution using Uniform Manifold Approximation and Projection (UMAP), performed by the umap R package. The pheatmap R package was used for the heatmap plot generation using the normalized expression data from the samples used in the study. Venn diagrams were generated via the online tool Venny 2.1.0 (https://bioinfogp.cnb.csic.es/tools/venny/index.html).

### miRNA functional characterization

The miRNAs categorized as upregulated and downregulated from each comparison were subjected to over-representation analysis (ORA) to understand the Kyoto Encyclopedia of Genes and Genomes (KEGG) pathways enriched for the corresponding targets using the miRNA Enrichment Analysis and Annotation Tool mieAA 2.0 web server (https://ccb-compute2.cs.uni-saarland.de/mieaa2/). The parameters used include an FDR (Benjamini–Hochberg adjustment) threshold of < 1, with minimum hits > = 2, and a background set to include only the miRNAs present in the NanoString Human v3A miRNA panel. Furthermore, only the KEGG pathways found to be enriched with a p-value for individual enrichment of < 0.05 were studied. If only a single miRNA satisfied the thresholds used for classifying miRNA as upregulated or downregulated, then for the purpose of ORA we considered all the miRNAs as having a positive (or negative) FC value and a p-value of < 0.05 along with a normalized expression within either of the two groups being compared > the average of negative control probe counts, in order to understand the major pathways being regulated.

Gene set enrichment analysis (GSEA) was performed on each individual comparison using all the miRNAs present in the nCounter Human v3 miRNA Panel using RbiomirGS on REACTOME pathways, downloaded from MSigDB database v.2022.1. (https://www.gsea-msigdb.org/gsea/msigdb/collections.jsp). The predicted miRNA to mRNA interactions were obtained from the validated mRNA target databases and combined with data from the miRNA differential expression analysis (estimated FC and p-values for each miRNA). All miRNAs were then given an SmiRNA score -log10 *P*-value * sign(log2FC), and for each mRNA, an SmRNA score was calculated by summing up the SmiRNA scores of all the predicted miRNA to mRNA interactions. The SmRNA scores were then used to perform logistic regression, which provides the likelihood of gene sets being more or less suppressed due to differential expression of miRNAs.

### miRNA-mRNA-pathway interactome

For every comparison, we performed an interactome visualization using the interaction between the significantly upregulated and downregulated miRNAs and their top five target mRNAs. The major pathways being enriched within the differentially expressed miRNAs in each comparison were also added to the interactome. Briefly, the target mRNA prediction for all upregulated and downregulated miRNAs was performed using MIENTURNET against the miRTarBase database with the following parameters: minimum number of interactions - 3, FDR <= 0.5, and a p-value <= 0.05. A further top five targets were chosen for each miRNA on the basis of highly significant unique p-values for the interaction between the miRNA and mRNA. The top targets of the upregulated and downregulated miRNAs were analyzed separately for the REACTOME pathway enrichment using the web-based DAVID v.2021 (https://david.ncifcrf.gov/) tool. The upregulated and downregulated miRNAs, their top mRNA targets, and the pathways enriched with a p-value of < 0.05 were visualized as an interactome using Cytoscape v3.9.1.

All statistical analysis and visualizations were performed using R Statistical Software v.3.6.1.

## Results

### Basic characteristics

Our study utilized 24 samples for miRNA profiling assay from HC (n=6; no of males- 2 and females-4), LTB (n=6; no of males- 3 and females-3), DS-TB (n=6; no of males- 5 and females-1) and DR-TB (n=6; no of males- 5 and females-1) participants. The median age of the participants was 33.5 (Range:19-44) for the HC group, 31 (Range:22-38) for the LTB group, 29 (Range:19-44) for the DS-TB group, and 32 (Range:18-47) for the DR-TB group. The demographics of the study population is represented in **Table 1**. Only two groups, HC and DR-TB, underwent a reduction in sample size as two samples from each group were filtered owing to poor ligation QC values below the threshold. The final number of samples used for further analysis was 20 samples.

**Table 1:** Demographics of the study population.

Groups	HC (n=6)	LTB (n=6)	DS-TB (n=6)	DR-TB (n=6)
Gender (Male/Female)	2/4	3/3	5/1	5/1
Age (Median, IQR)	33.5 (19-44)	31 (22-38)	29 (19-44)	32 (18-47)
IGRA status	Negative	Positive	–	–
Smear/Gene Xpert	–	–	Positive	Positive
Drug Sensitivity/Resistance	–	–	Sensitive	Resistant

**Figure 1:**
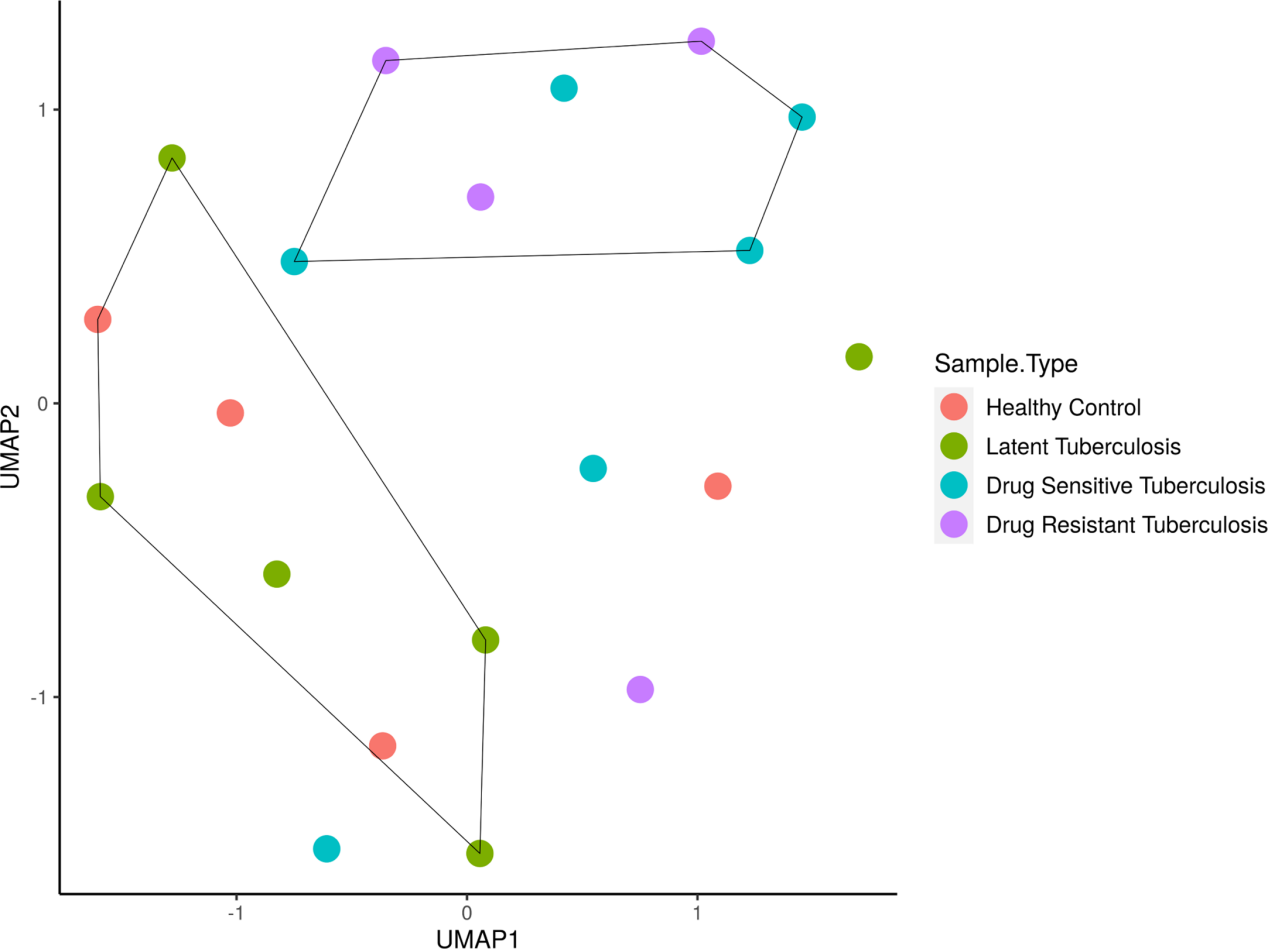
Sample clustering based on the normalized global miRNA expression visualized as a UMAP cluster plot. The samples are stratified globally into two groups; HC and LTB in one group and DS-TB and DR-TB in another group. *UMAP, Uniform Manifold Approximation and Projection for dimension reduction; HC, Healthy controls; LTB, Latent Tuberculosis; DS-TB, Drug- sensitive tuberculosis; DR-TB, Drug-resistant tuberculosis.

The sample distribution and clustering were visualized using their global miRNA expression profile through the dimensional reduction algorithm of uniform manifold approximation and projection (UMAP) (**Figure 1**). Based on the phenotypic similarities, the samples were observed as two clusters globally, where the LTB and HC samples clustered close to one another due to their healthy status while the active disease (DR-TB and DS-TB) samples formed another group. However, it was seen that owing to the heterogeneity within the miRNA expression profiles, certain samples from each group did not cluster within these healthy and active disease groups.

### Differential expression of monocyte miRNAs

The miRNA NanoString panel used for our study consisted of 799 miRNAs, of which, our differential expression analysis resulted in 107 statistically significant miRNAs across the various groups of comparison. The study group comparisons and the DE miRNAs compared between them are listed as follows: LTB vs HC: one miRNA (one upregulated); DS-TB vs HC: 17 miRNAs (one upregulated and 16 downregulated); DS-TB vs LTB: 26 miRNAs (two upregulated and 24 downregulated); DR-TB vs HC: 37 miRNAs (four upregulated and 33-downregulated); DR-TB vs LTB: 62 miRNAs (three upregulated and 59 downregulated); and DR-TB vs DS-TB: four miRNAs (two upregulated and two downregulated). The differentially expressed (DE) miRNAs (DEMs) (upregulated and downregulated) across different group comparisons are shown in **Table 2**.

**Table 2:** Differentially expressed miRNAs (upregulated and downregulated) across various comparisons.

Group Comparisons	Differentially expressed* miRNAs	Upregulated* miRNAs	Downregulated* miRNAs
LTB vs HC	1	1 miR-486-3p	–
DS-TB vs HC	17	1 miR-132-3p	16 miR-512-3p, miR-219a-1-3p, miR-506-5p, miR-494-5p, miR-1271-5p, miR-1299, miR-193b-3p, miR-499a-3p, miR-1288-3p, miR-198, miR-1245b-5p, miR-520c-3p, miR-370-5p, miR-455-5p, miR-485-3p, miR-1976
DS-TB vs LTB	26	2 miR-4516, miR-132-3p	24 miR-146a-5p, miR-6511a-5p, miR-486-3p, miR-617, miR-1915-3p, miR-1306-5p, miR-342-3p, miR-942-3p, miR-506-5p, miR-409-3p, miR-370-5p, miR-892a, miR-221-5p, miR-1226-3p, miR-512-3p, miR-517b-3p, miR-492, miR-1245b-5p, miR-877-5p, miR-491-3p, miR-326, miR-376a-3p, miR-455-5p, miR-136-5p
DR-TB vs HC	37	4 miR-199a-3p + miR-199b-3p, miR-1277-3p, miR-132-3p, miR-150-5p	33 miR-378b, miR-1224-5p, miR-1298-5p, miR-34b-3p, miR-548m, miR-1299, miR-563, miR-550a-5p, miR-1185-5p, miR-650, miR-499a-3p, miR-196a-3p, miR-1295a, miR-548ak, miR-548i, miR-219a-1-3p, miR-548a-5p, miR-337-5p, miR-365b-5p, miR-561-5p, miR-631, miR-182-3p, miR-6503-5p, miR-1250-5p, miR-2682-5p, miR-885-3p, miR-329-5p, miR-376c-5p, miR-5010-5p, miR-571, miR-337-3p, miR-504-3p, miR548z+miR-548h-3p
DR-TB vs LTB	62	3 miR-199a-3p + miR-199b-3p, miR-132-3p, miR-150-5p	59 miR-378b, miR-1183, miR-563, miR-548m, miR-339-3p, miR-675-5p, miR-6503-3p, miR-1908-3p, miR-664b-5p, miR-629-5p, miR-3196, miR-4284, miR-371b-5p, miR-376c-5p, miR-499a-3p, miR-508-5p, miR-1249-5p, miR-483-3p, miR-941, miR-1226-3p, miR-455-3p, miR-650, miR-548ak, miR-26a-5p, miR-550a-5p, miR-555, miR-365b-5p, miR-297, miR-1295a, miR-504-3p, miR-129-2-3p, miR-492, miR-589-5p, miR-518b, miR-486-3p, miR-329-5p, miR-196a-3p, miR-433-5p, miR-142-5p, miR-582-5p, miR-367-3p, miR-10b-5p, miR-337-5p, miR-885-3p, let-7g-5p, miR-3180, miR-151a-5p, miR-182-3p, miR-548i, miR-1306-5p, miR-5010-3p, miR-181b-2-3p, miR-888-5p, miR548z+miR-548h-3p, miR-101-3p, miR-146b-5p, miR-300, miR-337-3p, miR-92b-3p
DR-TB vs DS-TB	4	2 miR-451a, miR-150-5p	2 miR-631, miR-548m

* Threshold used for filtering differentially expressed or upregulated and downregulated miRNAs include, p-value < 0.05 and fold change >= 1.5 or <= -1.5.

**Figure 2:**
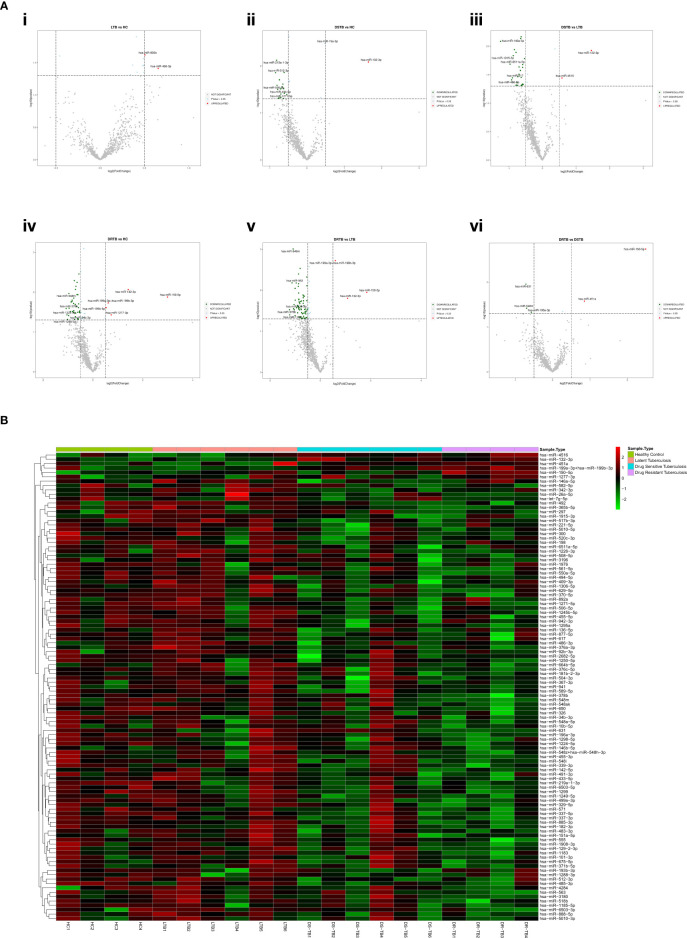
Differentially expressed (DE) miRNA signature on the TB disease spectrum. **(A)** Volcano plots of various study group comparisons. Cut-off points of log 2-fold change (<-0.5 and >0.5) and -log 10 p-value (1.3) are defined by grey dotted lines. The top 5 up and downregulated miRNAs are mentioned in the plots. **(B)** One-way hierarchical clustering of all 107 DE miRNAs across the samples of HC, LTB, DS-TB, and DR-TB groups. The upregulation is shown in the red color and the downregulation is shown in the green color.

Based on the log 2-FC and -(log10(p-value)), the top five upregulated and five downregulated miRNAs were delineated by volcano plots (**Figure 2A**). The top-most upregulated and downregulated miRNA for each comparison are as follows: LTB vs HC: miR-486-3p (up); DS-TB vs HC: miR-132-3p (up) and miR-512-3p (down); DS-TB vs LTB: miR-132-3p (up) and miR-146a-5p (down); DR-TB vs HC/LTB: miR-150-5p (up) and miR-378b (down); and DR-TB vs DS-TB: miR-150-5p (up) and miR-631 (down).

### miRNA clustering reveals the heterogenous response of DS-TB samples

The one-way clustering of all 107 statistically significant miRNAs resulting from all 6 group comparisons (**Figure 2B**) showed the heterogenous expression of these miRNAs across all the samples, particularly in the DS-TB group. The homogenous response was seen to be lost among the samples of the DS-TB group since they presented varied miRNA expression with the response being high in some samples and, in some samples, it remained low. In the case of LTB, one sample showed abnormal expression. However, the homogeneity was maintained in the DR-TB samples as they almost had a fairly uniform expression of miRNA across all four samples.

### Downregulation of monocyte miRNAs in active disease

**Figure 3:**
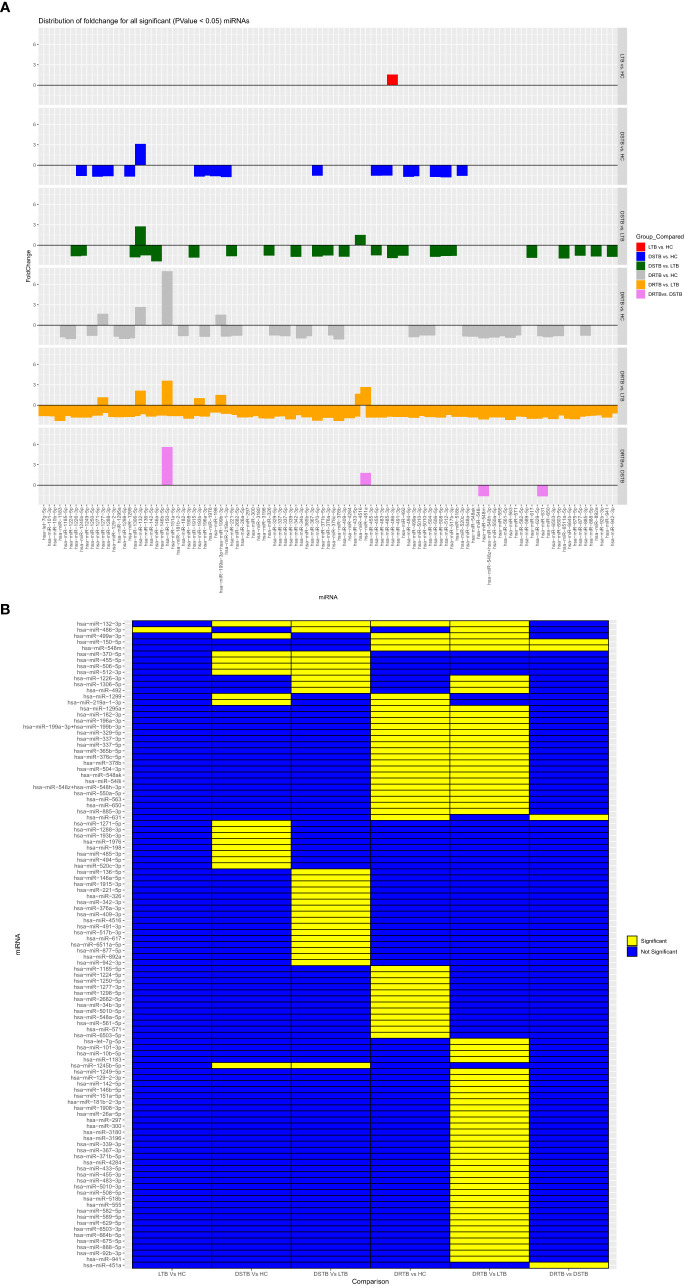
Fold change distribution **(A)** and statistical significance **(B)** of all 107 differentially expressed miRNAs across various study group comparisons. Lane 1: LTB vs HC, Lane 2: DS-TB vs HC, Lane 3: DS-TB vs LTB, Lane 4: DR-TB vs HC, Lane 5: DR-B vs LTB, and Lane 6: DR-TB vs DS-TB for **(A)** and in the same order from column 1-6 for **(B)**.

**Figure 4:**
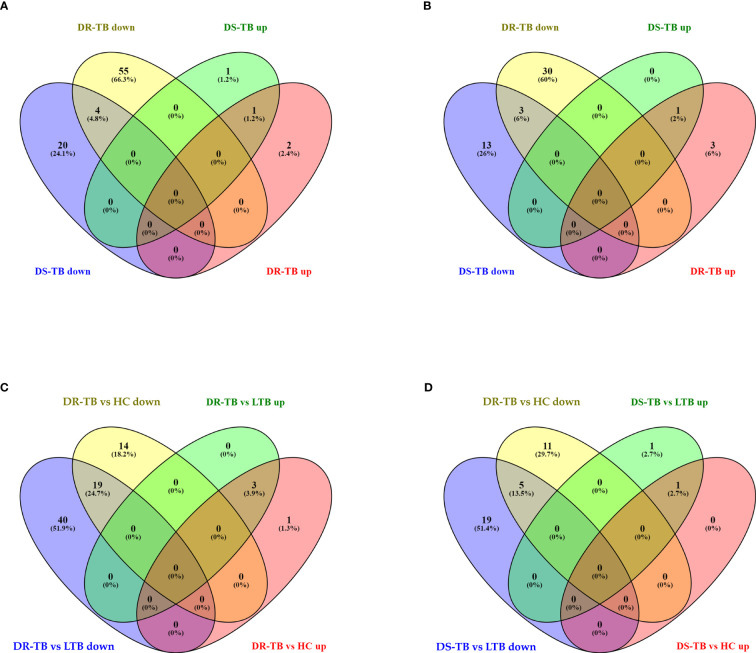
Shared and unique up and downregulated miRNAs of active disease versus latent and healthy status. **(A)** Active disease (DR-TB and DS-TB) versus LTB, **(B)** Active disease versus HC, **(C)** DR-TB versus LTB and HC, and **(D)** DS-TB versus LTB and HC. *Upregulated (up) and Downregulated (down).

The FC distribution and statistical significance of the 107 DE miRNAs across various group comparisons (LTB vs HC, DS-TB vs HC, DS-TB vs LTB, DR-TB vs HC, DR-TB vs LTB, and DR-TB vs DS-TB) are represented in **Figures 3A**, **3B**. As seen in **Figure 3A**, there was a substantial downregulation of multiple miRNAs in the active disease (both DR-TB and DS-TB) compared to LTB and healthy individuals. The shared and unique miRNAs expressed in DR-TB and DS-TB compared to LTB and HC are represented in **Figure 4**. Both DR-TB and DS-TB shared a significant decrease of four miRNAs (miR-1226-3p, miR-1306-5p, miR-486-3p, and miR-492) compared to LTB with FC ranges from -1.89 to -1.55 (**Figure 4A**) and three miRNAs (miR-1299, miR-219a-1-3p, and miR-499a-3p) compared to the HC (**Figure 4B**) group with FC values of -1.93 to -1.62.

Among the downregulated miRNAs, miR-548m and miR-631 were remarkably decreased in DR-TB alone compared to all other groups. Of these, the decrease of miR-548m in DR-TB was statistically significant when compared to DS-TB (FC: -1.62, p = 0.04), LTB (FC: -2.11, p < 0.05), and HC (FC: -1.94, p = 0.01), whereas the decrease of miR-631 showed a statistical significance in comparison with DS-TB (FC: -1.64, p = 0.01) and HC (FC: -1.62, p = 0.008) and not with LTB. DR-TB exhibited a significant decline of 19 miRNAs (miR-1295a, miR-182-3p, miR-196a-3p, miR-329-5p, miR-337-3p, miR-337-5p, miR-365b-5p, miR-376c-5p, miR-378b, miR-499a-3p, miR-504-3p, miR-548ak, miR-548i, miR548m, miR-548z + miR548h-3p, miR-550a-5p, miR-563, miR-650, and miR-885-3p) compared to the LTB and HC groups with FC ranges from -1.5 to -2.33 (**Figure 4C**). The miRNA signatures (five miRNAs) was significantly downregulated in DS-TB compared to LTB and HC were miR-1245b-5p, miR-370-5p, miR-455-5p, miR-506-5p, and miR-512-3p (**Figure 4D**) with FC values of -1.5 to -2.33.

However, the miRNAs upregulated in active disease included miR-1277-3p, miR-132-3p, miR-150-5p, miR-199a-3p + miR-199b-3p, miR-4516, and miR-451a. Both DR-TB and DS-TB shared a significant elevation of miR-132-3p compared to LTB (DR-TB vs LTB- FC: 2.12, p = 0.02 and DS-TB vs LTB- FC: 2.75, p = 0.01) and HC (DR-TB vs HC- FC: 2.67, p = 0.01 and DS-TB vs HC- FC: 3.12, p = 0.01) groups. Particularly, miR-150-5p was tremendously increased in DR-TB alone compared to all other groups (DR-TB vs DS-TB- FC: 5.61, p = 0.002; DR-TB vs LTB - FC: 3.59, p = 0.01; and DR-TB vs HC- FC: 7.93, p = 0.01). In addition, DR-TB exhibited a significant increase of miR-451a compared to DS-TB (FC: 1.8, p = 0.03). When compared to the LTB and HC groups, three miRNAs (miR-132-3p, miR-150-5p, and miR-199a-3p + miR-199b-3p) were significantly increased in the DR-TB group with FC values ranging from 1.51 to 7.93 (**Figure 4C**) and miR-1277-3p had a significant increase in DR-TB when compared to HC alone (FC: 1.66, p = 0.04). Likewise, DS-TB showed a significant increase of miR-4516 when compared with LTB alone (FC: 1.5, p = 0.04).

One miRNA (miR-486-3p) was bidirectional between active disease (DR-TB and DS-TB) (down) and latent infection (up) and had a significant reduction in DR-TB (FC: -1.7, p = 0.03) and DS-TB (FC: -1.89, p = 0.04) compared to LTB and not with HC while there was a significant elevation in LTB ((FC: 1.57, p = 0.04) when compared with HC.

### Over-representation analysis for KEGG pathways of up and downregulated miRNAs

We performed an over-representation analysis to understand the major KEGG pathways mediated by the upregulated and downregulated miRNAs resulting from each comparison. It was observed from the ORA that some of the significant TB-associated functions were enriched within the upregulated and downregulated miRNA set from various comparisons (**Supplementary Files 1**, **2**). The upregulated miRNAs of two important group comparisons (LTB vs HC and DR-TB vs DS-TB) showed the enrichment of significant pathways. Of these, the upregulated miRNAs of LTB vs HC regulate antigen processing and presentation, Th1 and Th2 cell differentiation, inflammatory mediator regulation of TRP channels, cytosolic-DNA sensing pathway, and mitophagy in animals. For DR-TB vs DS-TB, the only enriched function by the upregulated miRNAs was tyrosine metabolism. In addition, the upregulated miRNAs of DR-TB and DS-TB compared to LTB and HC were found to enrich certain significant pathways. Among them, the elevated miRNAs of DS-TB vs LTB did not represent enriched pathways whereas DR-TB vs LTB were found to mediate ECM-receptor interaction and peroxisome. The upregulated miRNAs of DR-TB vs HC mediated pathways such as hematopoietic cell lineage and biosynthesis of unsaturated fatty acids while those of DS-TB vs HC regulated pathways related to glycosphingolipid biosynthesis: lacto and neolacto series, hippo signaling pathway, and complement and coagulation cascades.

The downregulated miRNAs of an important comparison (DR-TB vs DS-TB) were not found to have significant functional enrichment. However, the downregulated miRNAs of both DR-TB and DS-TB had significant enrichment when compared to the LTB and HC groups. Some of the TB-associated functions mediated by the downregulated miRNAs of active disease versus latent infection are as follows: i) DS-TB vs LTB: TGF-beta signaling pathway and mTOR signaling pathway; and ii) DR-TB vs LTB: Wnt signaling pathway, calcium signaling pathway, and hedgehog signaling pathway. The enriched functions for the active disease versus the healthy controls are i) DS-TB vs HC: VEGF signaling pathway, oxidative phosphorylation, and Fc gamma R-mediated phagocytosis and ii) DR-TB vs HC: inflammatory mediator regulation of TRP channels, chemokine signaling pathway, apoptosis-multiple species, C-type lectin receptor pathway, cytosolic-DNA sensing pathway, and phospholipase D signaling pathway. These analyses help to define the functional pathways mediated by the up and downregulated miRNAs.

### GSEA revealed the bidirectional response of active disease and latent infection

**Figure 5:**
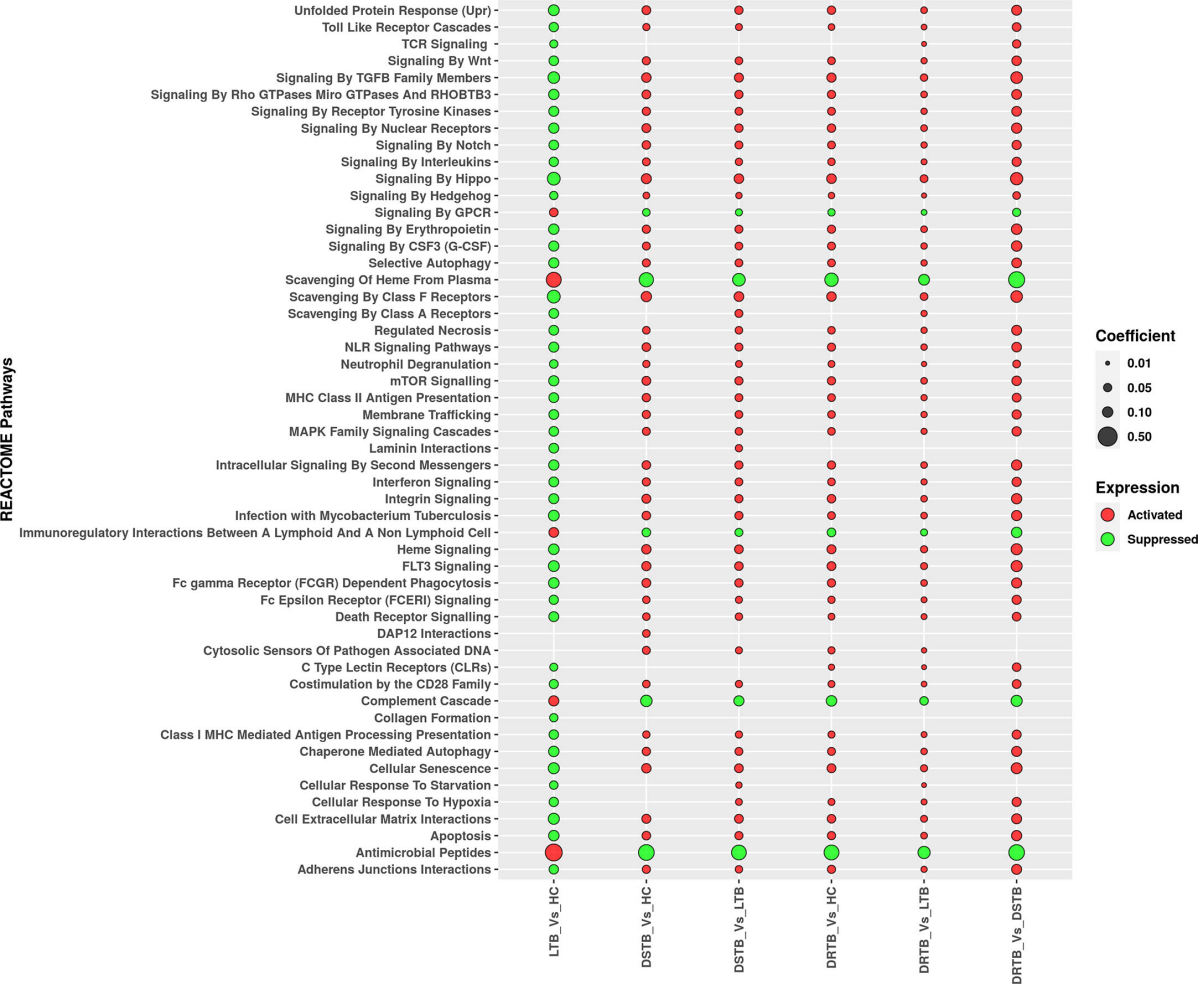
Regulation of major REACTOME pathways defined by miRNA gene set enrichment analysis. Activated functions are represented in red bubbles and suppressed functions are represented in green bubbles. The coefficient value defines the size of the bubble.

We employed GSEA to understand the directionality of regulation for the functions (REACTOME pathways) mediated by the miRNAs of the various comparisons. The status of activation or suppression of pathways among different comparisons (LTB vs HC, DS-TB vs HC, DS-TB vs LTB, DR-TB vs HC, DR-TB vs LTB, and DR-TB vs DS-TB) was represented as a bubble plot (**Figure 5**). The functions under the class of autophagy, programmed cell death, immune system, signal transduction, cellular responses to stimuli, extracellular matrix organization, cell-cell communication, and vesicle-mediated transport were considered in particular for the GSEA bubble plot in order to comprehend their modulation during TB disease spectrum (**Supplementary File 3**). The active disease displayed the activation profile when compared with LTB and HC. Specifically, DR-TB exhibited the hyperactivation of almost all the functions compared to DS-TB. The coefficient value describes the top three functions of DR-TB vs DS-TB such as signaling by hippo, signaling by TGF-β family members, and scavenging by class A receptors. As expected, the direction of the activated functions in DR-TB and DS-TB was opposite in LTB when compared to HC.

In contrast, the activated functions of latent TB vs HC were severely suppressed in active disease (DR-TB and DS-TB) compared to LTB and HC. The miRNAs from the LTB vs HC comparison were found to mediate the activation of antimicrobial peptides, scavenging of heme from plasma, complement cascade, immunoregulatory interactions between a lymphoid and a non-lymphoid cell, and signaling by GPCR. Thus, a bidirectional response was observed between active disease and latent infection with respect to the directionality of the functions mediated by miRNAs.

### miRNA-mRNA-pathway interactome visualization

**Figure 6:**
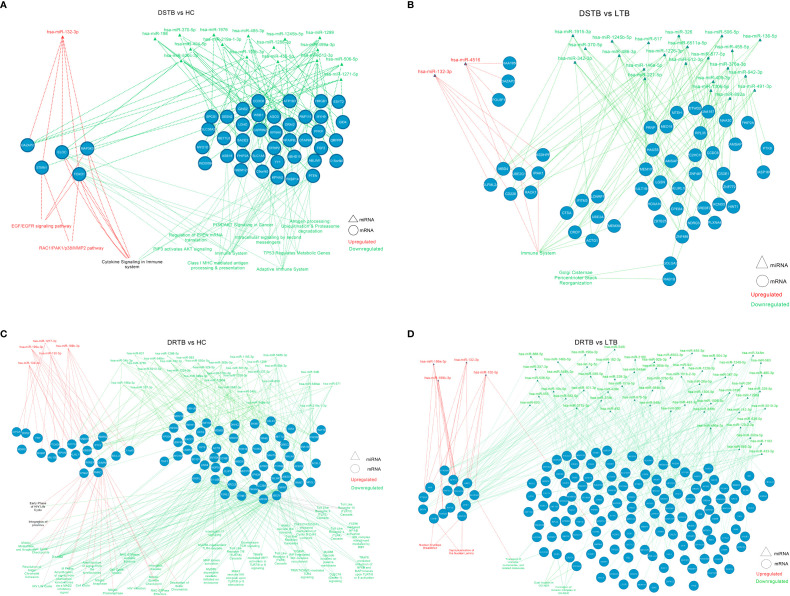
miRNA-mRNA-pathway interactome of top mRNAs targeted by up and downregulated miRNAs of active disease versus latent and healthy condition. **(A)** DS-TB vs HC, **(B)** DS-TB vs LTB, **(C)** DR-TB vs HC and **(D)** DR-TB vs LTB. Upregulated miRNAs and their targeted pathways are defined in the red color and downregulated miRNAs and their targeted pathways are defined in the green color. The pathways shown in black are targeted by targets of both upregulated and downregulated miRNAs.

The significantly differentially expressed miRNAs from each comparison were connected with their top five targets and pathways and visualized further as a miRNA-mRNA-pathway interactome (**Supplementary Files 4**, **5**). The miRNA-mRNA-pathway interactome for various comparisons is represented in **Figure 6**. The top mRNAs of both the upregulated and downregulated miRNAs of important group comparisons (LTB vs HC and DR-TB vs DS-TB) did not show significant enrichment of the functional pathways as expected and hence their interactome was not visualized. Similar to this, the comparison between active disease and latent infection (DS-TB vs LTB and DR-TB vs LTB) generated some functions that were statistically significant but did not share a major association with TB (**Figures 6B, D**). One exception was observed in the downregulated miRNAs and the top mRNAs of DS-TB vs LTB, where they showed interaction with immune system function.

However, the comparison between active disease versus healthy controls (DS-TB vs HC, and DR-TB vs HC) yielded significant functions that are associated with monocytes during TB (**Figures 6A, C**). The regulated functions of the upregulated miRNAs and their top targets for DS-TB vs HC are the EGF/EGFR signaling pathway, cytokine signaling in the immune system, and the RAC1/PAK1/p38/MMP2 pathway, and for DR-TB vs HC, no pathways were significantly enriched.

The top mRNA targets for the downregulated miRNAs were found to regulate the following functions in the active disease samples compared to healthy controls are as follows: i) DS-TB vs HC: adaptive immune system, Class I MHC mediated antigen processing and presentation, antigen processing: ubiquitination and proteasome degradation, cytokine signaling in the immune system, and PIP3 activates AKT signaling; and ii) DR-TB vs HC: TICAM1 and IRAK1 mediated IKK induction and TLR4 signaling, TLR cascades (3, 4, 5, 7, 8 and 10), MyD88 pathway, IL-17 signaling, FCERI mediated NF-kB activation, downstream TCR signaling, CLEC7A (Dectin-1) signaling, and TRAF6 mediated IRF7, NFkB, and MAP kinase upon TLR 7/8/9 activation. Hence, the miRNA-mRNA-pathway interactome provides an overview of the major pathways associated with top mRNAs targeted by the up and downregulated miRNAs.

## Discussion

Towards achieving the global end of TB strategy, scientific communities are working on multiple components in parallel to understand the disease behavior that could pave the road for advancement in diagnostic and therapeutic utilities. In this regard, the last decade of scientific research has unveiled miRNAs as the intrinsic mediator of protective and pathological responses (33, 34) in human monocytes or macrophages upon MTB infection. At the molecular level, MTB induces an alteration of miRNA levels that could seize cell differentiation and tune the macrophage responses for their survival (31, 35). Although the phenotypic changes of monocytes are well documented, fewer studies are conducted regarding their differences or how their transcriptome is altered. Previously, we have studied and reported the phenotypic differences of the monocyte subsets across the TB disease spectrum and found increased frequencies of intermediate monocyte subsets in active disease (DR-TB and DS-TB) compared to latent infection (36). To further understand the disease pathology, we have explored the transcriptional response of sorted monocytes mediated through miRNAs by profiling them via NanoString technology. The outcome was the differential expression of 107 miRNAs, particularly the downregulation of miRNAs in active TB groups (both drug-resistance and drug-sensitive). The miRNA profile revealed differential expression signatures: i) decline of miR-548m in DR-TB alone, ii) decline of miR-486-3p in active TB but significant elevation only in LTB, iii) elevation of miR-132-3p in active TB, and iv) elevation of miR-150-5p in DR-TB alone.

The upregulated miRNA signature (miR-150-5p and miR-451a) of DR-TB compared to DS-TB have been identified for their regulatory effects during TB with inconsistent expression patterns. In previous reports on TB, miR-150-5p is significantly downregulated in PBMCs, PBMC-derived macrophages, leukocytes, and blood of pulmonary tuberculosis (PTB) patients compared to LTB (37, 38) and HC (39, 40). miR-150 was considered to be a cell differentiation regulator of B cells, T cells, and NK cells, and its lower expression was correlated with the reduced mature forms of above mentioned cells (38, 40–42). The inverse association of miR-150-5p (up) and the β-arrestin 2 gene (ARBB2) (down) in LTB compared to PTB was found to regulate the immune responses to MTB (37). The reduced miR-150 from PBMC-derived macrophages of PTB was predicted to be involved in Wnt signaling, insulin signaling, TGFβ signaling, and glycosaminoglycan biosynthesis (38). Further, Wang et al. describe the decrease of miR-150-5p in serum samples of DR-TB compared to PTB and HC (43). Interestingly, our monocytes exhibited a tremendous rise of miR-150-5p in DR-TB compared to all other groups. This suggests that monocytes from the DR-TB group have a different miRNA expression profile that could probably be involved in drug resistance and disease pathogenesis. In the case of miR-451a, previous studies on the PTB group exhibited mixed expression of both increased and decreased expressions compared to LTB (44–46). Our study revealed the elevation of miR-451a in active TB (DR-TB and DS-TB) compared to LTB and an increase in DR-TB vs DS-TB. Studies have postulated that miR-451, along with miR-144, mediate erythroid homeostasis (47) and their augmented levels in active TB may lead to an altered immune response by modulating the immune cell profile (46). From our current study, tyrosine metabolism was the only function identified to be enriched for the upregulated miRNA profile (miR-150-5p and miR-451a) in the DR-TB versus DS-TB comparison through ORA. However, this has not been previously reported elsewhere.

Our study had one unique miRNA (miR-486-3p) that showed bidirectional expression between active disease (downregulated in DR-TB vs LTB and DS-TB vs LTB) and latent infection (upregulated in LTB vs HC). This was contradictory, as previous TB studies reported the elevation or decrease of 5-prime miR-486 (i.e. miR-468-5p) in PTB compared to LTB or HC (45, 46, 48). The role of miR-486-5p was suggested to regulate the genes of the nuclear factor of activated T cells (NFAT5) pathway (48). This further reaffirms that monocytes have different miRNA expression profiles compared to PBMCs or T cells as the 3-prime end of miR-486 was more predominant than the 5-prime in all TB-infected groups and thus signifies their pathological behavior. 

One miRNA that was remarkably increased in active TB (DR-TB and DS-TB) compared to LTB and HC was miR-132-3p. There was evidence for the increased expression of miR-132 in the MTB-infected cells versus the uninfected cells (49) and as a biomarker (50) for PTB. miR-132/miR-212 are involved in regulating TLR-2 mediated tolerance by targeting IRAK4 (51). In TB, MTB survival is favored by upregulated miR-132 and miR-26a via targeting p300 that predominantly decreases the host-mediated IFN-γ activation and phagocytosis (49). This postulates that increased miR-132-3p expression in active TB assists in TB disease progression.

However, we have observed the downregulated miRNA signature of DR-TB (miR-548m and miR-631) compared to DS-TB for the first time. In one study, miR-631 was overexpressed in the sputum of TB patients compared to controls but its definitive role is unknown (52). Moreover, these miRNAs did not have enriched functions in the ORA of DR-TB vs DS-TB from our results. Indeed, these miRNAs showed a protective response during cancer studies where decreased miR-548m expression suppresses cell migration and invasion by reversing the epithelial-mesenchymal transition of breast cancer via targeting aryl hydrocarbon receptor (53). Similarly, intrahepatic metastasis of hepatocellular carcinoma was inhibited by miR-631 by targeting the receptor-type protein tyrosine phosphatase epsilon (PTPRE) (54). This was quite surprising as these miRNAs appear to protect from cancer but aid immune activation and loss of protective balance in DR-TB.

Through systematic reviews, it is evident that miRNA expression (upregulation and downregulation) is inconsistent and heterogenous during TB disease because of various components such as age, gender, ethnicity, the technical platform used for profiling, and intrinsic biological characteristics (55, 56). We have demonstrated the substantial downregulation of monocyte miRNAs in active TB (DR-TB and DS-TB) with negligible upregulation compared to LTB and HC and this was even contradictory with miRNA expression from macrophages as reviewed previously by us (31). Collectively, our data show that these downregulated miRNAs of active TB were found to mediate mTOR signaling, TGF-β signaling, Wnt signaling, Hedgehog signaling, chemokine signaling, apoptosis, Fc gamma receptor-mediated phagocytosis, and oxidative phosphorylation with relevance to monocytic functions during TB disease. This downregulation is thought-provoking as both the ORA and miRNA-mRNA interactome (top mRNA functions) of the downregulated miRNA signatures from active TB were considerably better associated with the TB-related pathways than the upregulated miRNAs. Moreover, in our previous study, we suggested that reduced antigen presentation and processing was a result of diminished frequencies of dendritic cells due to impaired monocyte differentiation by modulated monocyte subsets in active TB (36). This may be true since the interactome of DS-TB vs HC revealed the downregulated miRNAs and their targeted top mRNAs mediated antigen processing and presentation. Another notable observation from the interactome was that cytokine signaling function in DS-TB, compared to HC, was mediated by the top mRNA targets of both upregulated and downregulated miRNAs. Recently, we reported a serial increase of circulating cytokines and chemokines from LTB to DS-TB to DR-TB (57, 58) and this was probably mediated by monocyte miRNAs as evidenced by the interactome of the current study. On top of it, IL-17 signaling was one of the predominant functions mediated by the top mRNA targets of downregulated miRNAs in DR-TB compared to HC. This could hint that monocyte miRNAs are the possible mediators of cytokine responses, particularly IL-17, evidenced by their hyper elevation in the DR-TB group as reported earlier (57).

On the whole, active TB exhibited differential expression and promising functions compared to a latent or healthy status. But the differences observed for LTB vs HC and DR-TB vs DS-TB were still negligible. As evidenced from the literature, the healthy controls and LTB fall under one end of the spectrum, where there are no radiological, microbiological, or clinical manifestations/confirmations, host immunological response alone can be helpful in distinguishing both categories. Similarly, the differences between DR-TB and DS-TB could be appreciated only through their treatment outcome. In our study, we have obtained more than five p-value significant miRNAs between these closely related groups (LTB vs HC and DR-TB vs DS-TB), but such miRNAs were not considered further due to the stringent criteria and were filtered based on FC cut-off (data not shown). The directionality of miRNA functions and the outcomes from miRNA GSEA overpowered these observations and facilitated two phenomenal findings: i) bidirectional response between active disease (activation profile in DR-TB and DS-TB compared to LTB and HC) and latent infection (suppression profile in LTB vs HC), and ii) hyper immune activation in the DR-TB group compared to DS-TB. GSEA also described the severe suppression of antimicrobial peptides and complement cascades in active disease and activation in latent infection and so the protection against MTB was severely stalled in active TB. Beyond the miRNA expression profile, GSEA demonstrated the hyperactivation in DR-TB vs DS-TB and severe suppression in LTB vs HC and defined the clear biological differences that exist between those closely related groups. That these differences were somehow missed through their expression patterns may be due to several reasons such as i) stringent criteria (p-value <0.05 and fold-change <-1.5 or >1.5) for selecting the significant miRNAs, ii) phenotypic similarities between HC and LTB and between DS-TB and DR-TB, iii) miRNA being the intermediate component of mRNAs and proteins thus they could not capture those differences, and iv) smaller sample size.

Altogether these findings offer reasonable evidence to advocate monocyte miRNAs as the potential activators of multiple immune mechanisms in active TB and thus impair the downstream functions of monocytes and favor MTB survival and disease progression. However, the sole function of monocytes and their miRNAs for TB pathology can be strongly proposed only after performing similar transcriptomic studies for other cell types such as neutrophils and T cells. Other major limitations of our study are sample size and a cross-sectional study design which severely impede the utility of observed miRNAs in clinical settings. Further collaborative studies and validation through a real-time PCR with a larger sample size are crucial for these miRNA signatures to be utilized as biomarkers for the TB spectrum. miRNA mimic or knockdown studies in both *in-vitro* and *in-vivo* animal studies are highly essential for further understanding their therapeutic utility.

To conclude, the downregulation of monocyte miRNAs implicates their immune dysfunction in DR-TB and thus future explorative studies on monocytes could provide deeper insight for developing better diagnostic strategies and therapeutic utilities for DR-TB.

## Data availability statement

The datasets generated and analyzed are included in the article and additional files. NanoString RNA transcript data were deposited in the Gene Expression Omnibus (GEO) of NCBI under accession number GSE229020 with the following link: https://www.ncbi.nlm.nih.gov/geo/query/acc.cgi?acc=GSE229020.

## Ethics statement

The studies involving human participants were reviewed and approved by ICMR-National Institute for Research in Tuberculosis Ethical committee (NIRT, IEC 2015022). The patients/participants provided their written informed consent to participate in this study.

## Author contributions

Designed research studies: PS, UR, and RB. Conducted experiments: PS, LM, AJ, and MD. Data analysis and interpretation: PS, MM, AM, GR, and RB. Contributed towards clinical and instrumental resources: AN, SH, and GR. Wrote the manuscript: PS, MM, GR, UR, and RB. Manuscript review and editing: PS, MM, UR, GR, and RB. All authors contributed to the article and approved the submitted version.

## Supplementary material

Supplementary File 1 Over-representation analysis results for KEGG pathways of upregulated miRNAs from various group comparisons (LTB vs HC, DS-TB vs HC, DS-TB vs LTB, DR-TB vs HC, DR-TB vs LTB and DR-TB vs DS-TB).

Supplementary File 2 Over-representation analysis results for KEGG pathways of downregulated miRNAs from various group comparisons (LTB vs HC, DS-TB vs HC, DS-TB vs LTB, DR-TB vs HC, DR-TB vs LTB and DR-TB vs DS-TB).

Supplementary File 3 Results of miRNA-Gene set enrichment analysis (Reactome pathways) of various group comparisons (LTB vs HC, DS-TB vs HC, DS-TB vs LTB, DR-TB vs HC, DR-TB vs LTB, and DR-TB vs DS-TB).

Supplementary File 4 Top mRNA targets of upregulated miRNAs and their related enriched pathways from various group comparisons for miRNA-mRNA-pathway interactome visualization (LTB vs HC, DS-TB vs HC, DS-TB vs LTB, DR-TB vs HC, DR-TB vs LTB and DR-TB vs DS-TB).

Supplementary File 5 Top mRNA targets of downregulated miRNAs and their related enriched pathways from various group comparisons for miRNA-mRNA-pathway interactome visualization (LTB vs HC, DS-TB vs HC, DS-TB vs LTB, DR-TB vs HC, DR-TB vs LTB and DR-TB vs DS-TB).
